# Insects Used as Food and Feed: Isn’t That What We All Need?

**DOI:** 10.3390/foods9081003

**Published:** 2020-07-27

**Authors:** Victor Benno Meyer-Rochow, Chuleui Jung

**Affiliations:** 1 Agricultural Science and Technology Research Institute, Andong National University, Andong 36729, Korea; 2 Department of Genetics and Ecology, Oulu University, SF-90140 Oulu, Finland; 3 Department of Plant medicals, Andong National University, Andong 36729, Korea

**Keywords:** entomophagy, insect edibility, food shortage, acceptance, bio-active compounds, nutrients

## Abstract

This Special Issue of *Foods* explores different aspects of how insects can be used as a novel resource for food and feed. Some contributions deal with questions of acceptability and legality, others tackle problems related to innovative techniques in processing and marketing food, and yet another group of papers highlights the use of insects and their bio-active products in the context of promoting human health. The collective aim of the contributions by the researchers from at least 20 countries is to examine whether the use of insects—be it for food, feed, or therapeutic purposes—has a future. We conclude that positive aspects undoubtedly exist regarding the nutritional and pharmacological value of various insect species but that environmental and bio-functional issues could even outweigh the nutritional value of food insects.

## 1. Introduction

The terms “entomophagy”—a combination of the ancient Greek words “entomon” (notched anima) and “phagein” (eating of something specific)—and “insectivorous” and “insectivore”, which use a suffix of Latin origin (derived from “vorare”, to devour), have been examined and compared with each other by Evans et al. [1] with regard to their suitability and applicability in connection with humans. That study not only demonstrates the uncertainty in how to describe the consumption of insects by humans, whether habitual or accidental, but also highlights that the use of insects by humans as food and feed can be approached from different angles in different disciplines by experts from different locations asking different questions and using a variety of tools. This Special Issue of *Foods* is a case in point because authors located in at least 20 different countries have contributed their findings to it.

The papers chosen to form part of this Special Issue range from those addressing legal matters and questions of acceptability to those that are primarily concerned with the processing, chemical composition, and bioactivity of the insects. The range of papers in this issue underscores the fact that a greater understanding of the potential of insects as food and feed depends on interactions between fields and experts of both the humanities and the sciences. Whether, however, insects will ever be the “food of the future” is difficult to say. They were undoubtedly “food of the past” for our ancestors, and they still are a “food of the present” for many inhabitants of the world today [2,3,4,5].

In the past, insects were indeed a food item appreciated by humankind worldwide [6,7,8]. Somehow—and for reasons not completely understood but probably related to the spread of Christianity that forbade the consumption of insects with the exception of four species of locust, or the awareness of the fact that insects can be vectors of some fatal human diseases, the emotional separation from nature giving rise to entomophobia, and an increasingly greater variety of food stuffs reaching the consumer—the use of insects as human food became continually less popular. Our closest animal relatives, the monkeys, have been observed to actively collect insects and other arthropods in order to eat them—e.g., [9,10,11,12]—or, in the case of millipedes, to use them therapeutically [13].

Over the last 30 years, there has been a renewed interest in insects as human food, and the literature dealing with entomophagy and entomotherapy has become so vast that it is seemingly impossible to review it in its entirety. International conferences have begun to focus more and more on edible insects since the XVI International Pacific Science Congress in Seoul in August 1987 and the International Conference on Minilivestock in Beijing in September 1995, and the subsequent book by Maurizio Paoletti [14] brought this topic to a wider audience. Scientific publications, too many to mention, have appeared in the last 20 years or so, praising the advantages of an insect-based diet over a diet consisting of conventional meats like poultry and especially ruminants and highlighting the environmentally advantageous farming practices for mini-livestock such as insects over those for traditionally farmed animals [15,16,17]. Various edible insect species have had their farming potential assessed, their acceptability as a novelty food (or feed in animal husbandry and fish culture) examined, and their potential risk of carrying diseases or undesired microbes scrutinized. Nevertheless, there remain many open questions, which is why Special Issues such as this are still important.

Although insects should not be seen as a food item for humans merely to survive times of dietary hardship and periods of starvation [18], there is no way of denying that the global food security situation for the human population is becoming increasingly precarious and that food production worldwide has to increase by at least 50% to meet the demand by 2050 [19]. Despite the earlier reports of traditionally living people in different parts of the world engaging in entomophagy (mentioned above), none of these reports until Meyer-Rochow [20] had thought to link global food security with insects being more universally and extensively used as a possible and potent way to ease global food shortages. It was largely due to DeFoliart [21] and Van Huis et al. [22] that the idea was taken up and more widely promoted.

We now possess a considerable amount of information on the kinds of insect that serve as food to various people in the world; we know that most insects are nutritious, consist of valuable protein and easily digestible fatty acids, and contain important minerals and vitamins [23,24,25], and recommendations exist on how to breed the most lucrative species optimally [26,27]. However, gaps to be filled still exist with regard to the processing of cultured insects, the preservation of insect-based foods and their nutrients, the preparation of insect-containing dishes, the economics and marketing, and the potential of insects as suppliers of health-promoting drugs and medicines.

## 2. Results

In this Special Issue of *Foods*, Dorothy Nyangena and colleagues [28] stress that quality and safety concerns call for simple, actionable hazard control mechanisms. They tested the extent to which different processing methods affected the proximate composition and microbiological characteristics of the edible cricket *Acheta domesticus* and the grasshopper *Ruspolia differens*. The researchers concluded that traditional processing can alter the nutritional value of the insects but improves microbial safety.

Microbial safety was also an issue that the paper by Nils Grabowski et al. [29] dealt with. The authors pointed out that edible insects, like other foodstuffs, in order to guarantee consumer safety and product quality, should be covered by governmental regulations. Since nations vary with regard to their approaches concerning the inclusion of insects in foodstuffs and commercial farming, the situation is complex. This paper reviews the situation from a hygiene point of view and provides some worthwhile recommendations.

The Korean scientists Su-Jin Pyo and colleagues [30] focused on six species of traditionally used insects in Korea and demonstrated that, in particular, *Allomyrina dichotoma* and *Teleogryllus emma* showed considerable antioxidant activities. *Tenebrio molitor* and honeybee extracts, more so than the other species, exhibited strong haemolytic activities, suggesting that these insects not only possessed potential as a food source but could be used therapeutically as well.

The paper by Sampat Ghosh and colleagues [31] shows that drones and the developmental stages of identical species (in this case, the honeybee *Apis mellifera*), but representing different strains from widely different localities (here: “Buckfast” drones from Denmark and “Italian” drones from Korea) can differ with regard to their composition. Buckfast drones, for example, exhibited the highest antioxidant activity.

Results on the basis of data obtained from 310 Italian consumers were analyzed by Rocco Roma and colleagues [32] in order to ascertain the acceptability of edible insects as food or food supplements. Consumers fell into five groups and the acceptance of insects as feed material amongst them was widespread, but acceptance as a food item for humans was not. Reasons such as neophobia, disgust, assumed health risk, unfamiliarity, and socio-cultural backgrounds are thought to be responsible, although age-dependent differences were noticed.

Giulia Leni and co-workers [33] present a novel method tested on the lesser mealworm *Alphitobius diaperinus* that can be used in conjunction with other insect species to extract high quality protein for food and feed purposes. Enzymatic hydrolysis with *Bacillus licheniformis* for five hours resulted in a protein hydrolysate with a 15% degree of hydrolysis, oil-holding capacity, and excellent solubility. 

Da-Young Lee and colleagues [34], who investigated the extent to which freeze-dried mature larval silkworm powder (SMSP) affected ethanol-induced fatty liver disease in rats, found that mature larval silkworm powder reduced triglyceride levels by as much as 35% in the livers of the rats that had received daily doses of 25% ethanol (3 g/kg body weight) for 4 weeks. In ethanol-treated rats given SMSP, the serum levels of triglycerides and various other compounds were lower, suggesting that SMSP can protect against alcoholic liver disease.

In view of the fact that so-called mirror neurons can influence a person’s decisions and/or opinions, Meyer-Rochow and Kejonen [35] analyzed a list of commonly used idioms in the Finnish language that make reference to insects in the context of food and eating. The vast majority of these idioms referred to insects in a negative way, which could lead to or reinforce feelings of disgust and prevent people from trying insect-containing food items.

That the nutrient composition of mealworms can be influenced by fresh plant supplements in their diet during the growing phase of the insects was demonstrated by Changqi Liu and co-workers [36]. When mealworm larvae were reared on wheat bran enriched with carrot, orange, or red cabbage for four weeks, the larvae grew better and bigger, being 40–46% heavier than the controls that did not receive the food supplements.

Yang-Ju Son and colleagues [37] used mealworms to produce a defatted powder and mealworm oil. While the powder was rich in protein, minerals, and bioactive compounds and had a pleasant taste, the mealworm oil contained bioactive nutrients, especially gamma tocopherol, and had physicochemical properties that were similar to those of vegetable oil. Mealworm products contain antioxidants and glucosamine derivatives such as chitin and chitosan and possess anti-inflammatory properties.

Inhabitants of the island of Madagascar, as Joost Van Itterbeeck and colleagues [38] could show, have a long history of using especially insects belonging to the Orthoptera as a traditional food. Thirty-seven edible species were recorded. The insects were mostly eaten by humans as snacks and part of the main meal and were primarily collected by children. With the exception of *Brachytrupes membranaceus colosseus*, the insects were not marketed, and using insects as feed for animals was rare.

The microbiological and bioactive properties of powders made from *Acheta domesticus* and *Tenebrio molitor* were examined by Concetta Maria Messina and co-workers [39], who found a higher number of viable bacteria, mainly *Citrobacter* and Enterobacteriaceae, in mealworm flour than in cricket powder, which, on the other hand, contained more Porphyromonadaceae. The protein hydrolysates of both powders were rich in antioxidants.

To what extent an aversion to insects and rejection of insects as human food exists in different countries was studied by Mauricio Castro and Edgar Chambers [40] with a survey launched in 13 countries. Most of the 630 participants in the study were reluctant to accept insects and insect-containing food items, with the exception of respondents from Mexico and Thailand. Obviously, that educating consumers that food insects need not be unhygienic should be one goal in making food insects more widely acceptable, was their conclusion.

The latter is also the recommendation that came from the results obtained by Karin Wendin and colleagues [41], who could show that sensory attributes are a very important factor. Particle size, for example, had a great influence on the appearance and viscosity, colour and odour, as well as texture and taste of the insect-containing food but not the flavour. On the other hand, salt content did affect the taste and flavour. An addition of antioxidants reduced the colour change and rancidity in the food, thereby improving its acceptability.

An interesting test was carried out by Simone Mancini and psychology colleagues [42], who asked participants to evaluate by taste two identical bread samples, one labelled as having been supplemented with insect powder. In reality, however, both types of bread were identical. Even though the participants gave higher scores for flavour, texture, and overall liking to the insect-labelled bread, they nevertheless declared that they were less likely to consume the insect-labelled bread in the future, which suggests deep-seated feelings of insect rejection overriding sensory experience.

The idea that the acceptability of insects as a component of food items depends to a large degree on the way in which the edible insects are processed and presented but also on the packaging has been suggested before, but Wendin, Mårtensson, Djerf, and Langton [43] confirmed this through sensory analyses with probands. The results showed that following mixing and chopping (e.g., of mealworms), the particle size, oil content, salt content, and antioxidants influenced the product more than the storage time. Packed in Tetra Recart cartons, the product also stayed stable for at least 6 months at room temperature.

That there are still big gaps regarding the use and acceptability of insects as human food in various regions of the world has been shown in an interesting paper by Mozhui et al. [44]. In this beautifully illustrated investigation of entomophagic practices by the inhabitants of Nagaland (North-East India), the authors not only provide details on how the 106 edible species (representing nine orders of insects and 32 families) are obtained but also explain the various ways in which local residents turn the insects into delicious dishes.

An important aspect of processing insects as human food is tackled by the Malaysian/Thai team headed by Anuduang [45]. In their contribution they investigated the impact of a range of temperatures and durations on the quality of silkworm powder and found that shorter times of exposure to hot water and a low drying temperature preserve the antioxidant activities of the product best.

Jeong et al. [46] point out that despite their long history of utilization as food and pharmaceutical bioresources, the value of social wasps as a food resource is poorly appreciated and researched. Especially, not only are the larvae of the invasive *Vespa velutina nigrithorax* rich in nutrition and safe to eat but their saliva’s amino acid composition has been shown to be worthy of further study in the context of amino acid supplementation and exercise enhancement.

## 3. Discussion

Coming back to the original question as to whether insects as food and feed have a future, we conclude that there can be no doubt that the many positive aspects in terms of environmental issues, especially when commercially bred species are considered, will outweigh the fact that the nutritional value of food insects is not significantly greater than that of conventional foods [47]. Insects bred to replace or supplement feedstuffs required in fish culture, poultry, and other animal husbandry will encounter fewer acceptability problems than insects and insect products aimed at human consumers [48]. Moreover, although numerous species of insects have been used therapeutically in the past [49], modern chemical analytical and isolating techniques have made it possible to identify potent compounds in them, and, as also some papers in this issue have shown, insects and insect products with medicinal properties are likely to play an increasingly greater role in the future to combat infections and other maladies. 

The main advantages of food insects over conventional meat-supplying species are that the former can be reared on much less food and water and require significantly less space than the latter. Furthermore, the rate of reproduction of insects is considerably higher than that of conventional food animals, and a significantly greater proportion of an insect’s body weight is utilizable as food than is the case with regard to conventional meat animals. Most people and not just animal lovers would also find the “harvesting” of insects more acceptable than the slaughter of livestock. Finally, the so-called carbon footprint of farmed insects is deemed to be appreciably lower than that of farmed conventional food animals [15,16,17,22].

Obstacles to overcome are, on the one hand, the declining interest in edible insects by citizens of countries in which insects used to be consumed widely [50] and, on the other hand, the still widespread rejection of insect-containing foods by citizens of countries in which edible insects and insect-supplemented foods were not traditionally accepted but have now become available [51,52,53]. To understand what governs the selection and acceptance of edible insect species and what role taboos play in this has to be one important goal [54]; education, clever marketing, tapping into the trend of buying healthy food, and highlighting the traditional nutritional and medical benefits are additional goals that could help to make consumers consider insects as food [55,56]. Using catchy slogans such as “Forget about the pork and put a cricket on your fork” or “Mealworms and spaghetti is food that makes you happy” could also be expected to help!
